# Early-onset schizophrenia: an adolescent case report

**DOI:** 10.1192/j.eurpsy.2023.2226

**Published:** 2023-07-19

**Authors:** C. Alcalde-Diosdado Crespi, E. K. Alvarado Altuve, S. Cruz Bailén, I. Caparrós del Moral

**Affiliations:** Complejo Hospitalario de Jaén, Jaén, Spain

## Abstract

**Introduction:**

This is the case of a girl, aged 13, starting on 2021 with a first psychotic episode. Before this episode, her psychiatric history was an adjustment disorder because of scholar bullying, fully recovered before the onset of the current symptoms.

**Objectives:**

To describe an interesting case of early-onset psychosis.

**Methods:**

We have used the interviews with the patient and her profile in Diraya (the medical database software in Andalucía).

**Results:**

The first symptoms started 6 months before the first hospitalization, and consisted in mild behavioural disorders, with disobedience and rudenesses, which represented a significant change compared with the previous personality of the patient. 3 weeks before the first admission she abruptly started to experience disconnection, unmotivated laughs, decreaded academic performance and incoherent speech. Also, she showed motor symptoms, consisting in oral and right-hand stereotypies. Then, she was hospitalized in a Pediatric unit, in order to rule out organicity. The nuclear magnetic resonance showed an image suggestive of venous development anomaly, with no acute injuries. Her cerebral spinal fluid was widely studied, and all the results were negative, including: the technique of PCR for many virus and bacteria that can cause meningitis or encephalitis; a bacterial culture; a biochemical study; antineuronal antibodies; and a limbic encephalitis antibodies study. Besides, the blood count, the biochemistry, the gasometry and serology were also negative. No drugs were detected in the urinalysis. Once the organicity was ruled out, she was treated with Olanzapine and Diazepam, and destinated to my child and adolescent psychiatry unit. During the first hospitalization we observed that she looked very often to the mirror, showed soliloquies and took leaps. During the interviews she was desinhibited. She initiated a delusional speech, focused in sexual topics. She said that she’s had a baby in the future with his father, and talked a lot about things she had already made in the future. During this admission, we changed the treatment to Quetiapine and Valproate.

The second hospitalization was was done due to a lack of efficacy with the previous treatment and the presence of autolytic thoughts. We switched from Quetiapine to Aripiprazole. After a few days, she showed again a desinhibited behaviour, and kept the delusional speech, that now was more complex, refering that she had more than 20 babies, with many different men. After this we tried Lurasidone and suspended Aripiprazole, she showed a clinical improvement, at the cost of many side effects, though. So we finally changed to Clozapine, in combination with Gabapentin. Since she got clinical levels of clozapine, the delusions have been encapsulated.

**Conclusions:**

The differential diagnosis is set with an early-onset schizophrenia and a schizoaffective disorder. Obviously, the evolution of the sypmptoms in the following months and years will have the last word.

**Disclosure of Interest:**

None Declared

